# The 15N and 46R Residues of Highly Pathogenic Porcine Reproductive and Respiratory Syndrome Virus Nucleocapsid Protein Enhance Regulatory T Lymphocytes Proliferation

**DOI:** 10.1371/journal.pone.0138772

**Published:** 2015-09-23

**Authors:** Baochao Fan, Xing Liu, Juan Bai, Yufeng Li, Qiaoya Zhang, Ping Jiang

**Affiliations:** 1 Key Laboratory of Animal Diseases Diagnostic and Immunology, Ministry of Agriculture, College of Veterinary Medicine, Nanjing Agricultural University, Nanjing, 210095, China; 2 Jiangsu Co-innovation Center for Prevention and Control of Important Animal Infectious Diseases and Zoonoses, Yangzhou, China; College of Veterinary Medicine, CHINA

## Abstract

Porcine reproductive and respiratory syndrome virus (PRRSV) negatively modulates host immune responses, resulting in persistent infection and immunosuppression. PRRSV infection increases the number of PRRSV-specific regulatory T lymphocytes (Tregs) in infected pigs. However, the target antigens for Tregs proliferation in PRRSV infection have not been fully understood. In this study, we demonstrated that the highly pathogenic PRRSV (HP-PRRSV) induced more CD4^+^CD25^+^Foxp3^+^ Tregs than classical PRRSV (C-PRRSV) strain. Of the recombinant GP5, M and N proteins of HP-PRRSV expressed in baculovirus expression systems, only N protein induced Tregs proliferation. The Tregs assays showed that three amino-acid regions, 15–21, 42–48 and 88–94, in N protein played an important role in induction of Tregs proliferation with synthetic peptides covering the whole length of N protein. By using reverse genetic methods, it was firstly found that the 15N and 46R residues in PRRSV N protein were critical for induction of Tregs proliferation. The phenotype of induced Tregs closely resembled that of transforming-growth-factor-β-secreting T helper 3 Tregs in swine. These data should be useful for understanding the mechanism of immunity to PRRSV and development of infection control strategies in the future.

## Introduction

Porcine reproductive and respiratory syndrome virus (PRRSV) is an enveloped and positive-stranded RNA virus of the family Arteriviridae, and causes significant economic losses in the global swine industry [1]. Based on genetic and antigenic characteristics, two major genotypes of PRRSV, type 1 (European; prototype strain Lelystad) and type 2 (North American; prototype strain VR-2332), have been identified and share 55–70% nucleotide identity [2–5].

The major characteristics of PRRS include reproductive failure and respiratory disease that usually lead to compromised lung defense mechanisms, followed by secondary complications [6, 7]. PRRSV can cause a prolonged viremia phase and persist in pigs for long periods, primarily in tonsils and lymph nodes, after initial infection [8–11]. In addition, PRRSV infection results in severe functional impairment of cells of the monocyte/macrophage lineage [7] and suppression of natural killer cell-mediated cytotoxicity against PRRSV-infected porcine alveolar macrophages *in vitro* [12]. Moreover, PRRSV infections have a negative impact on the host immune system that leads to inadequate response to vaccination [13–15]. All data reveal that PRRSV can modulate the porcine immune system, particularly during the early stages after infection. However, the mechanisms involved in these unusual and delayed immune responses remain to be elucidated [16–18].

Regulatory T lymphocytes (Tregs), which are determined by coexpression of CD4, CD25 and forkhead box (Fox)p3 are responsible for controlling the immune response and maintaining homeostasis, suppressing or controlling the function of effectors and immunocompetent cells [19]. Tregs are classified as natural or induced types. Natural Tregs mainly control the immune response against autoantigens and induced Tregs are involved in the response to exogenous antigens [19, 20]. Tregs are implicated in several persistent or chronic viral infections in humans, and increased numbers are observed in several chronic infections, including human immunodeficiency virus, hepatitis C virus (HCV) and human cytomegalovirus infections [21–24]. According to the cytokines that they produce, inducible Tregs can be classified into several subtypes: (1) TR1 cells that secrete interleukin (IL)-10; (2) T helper (Th)3 cells that secrete transforming growth factor (TGF)-β; and (3) converted Foxp3^+^ Tregs [19, 25]. Inducible Tregs acquire their function following infection or exposure to other stimuli [19]. In pigs, the CD4^+^CD25^+^FoxP3^+^ Treg cells exhibiting suppressor activity by a variety of mechanisms have been identified [26].

Recent studies have demonstrated induction of Tregs during the early phase of infection in PRRS. Type 2 PRRSV strains induce Tregs proliferation and upregulate TGF-β production [27–30]. PRRSV N protein plays an important role in IL-10 production [31]. However, monocyte-derived dendritic cells (MoDCs) infected with type 1 PRRSV produce neither TGF-β nor Tregs [32]. In this study, we found that HP-PRRSV induced Tregs proliferation more strongly than C-PRRSV. By using synthetic peptides and reverse genetic methods, it was firstly found that the 15N and 46R residues in PRRSV N protein play an important role in Tregs proliferation.

## Materials and Methods

### Viruses and cells

HP-PRRSV strain BB0907 (GenBank no. HQ315835) used in this study was isolated in Guangxi Province, China, in 2009. C-PRRSV strain S1 (NCBI GenBank no. AF090173) was isolated from pigs with clinical signs of PRRS in Jiangsu Province in 1997. Marc-145 cells were maintained in Dulbecco’s Modified Eagle’s Medium (GIBCO) supplemented with 10% fetal bovine serum (FBS; GIBCO) containing 100 U/ml penicillin and 100 μg/ml streptomycin at 37°C with 5% CO_2_. Once the cytopathic effect was apparent, cell cultures were freeze–thawed twice and the lysates were centrifuged at 650 × *g* at 4°C for 20 min. The supernatant containing the virus was collected, titrated, and stored at –70°C.

### Synthetic peptides

The synthetic peptides listed in Table 1 were obtained from SBS Genetech. Peptides 1–16 overlapped by 11 amino acids (aa) covered the full length of N protein of BB0907. The aa sequences of peptides 3m, 7m and 12m were the same as those of N protein of S1. All Peptides were synthesized as white powder to > 93% purity, and were dissolved in PBS to a concentration of 1 mg/ml prior to experiments.

**Table 1:** Synthetic peptides used in this study.

No.	Position	Sequence
1	1–18	MPNNNGKQQKKKKGNGQP
2	8–25	QQKKKKGNGQPVNQLCQM
3	15–32	NGQPVNQLCQMLGKIIAQ
4	22–39	LCQMLGKIIAQQNQSRGK
5	29–45	IIAQQNQSRGKGPGKKN
6	35–52	QSRGKGPGKKNRKKNPEK
7	42–59	GKKNRKKNPEKPHFPLAT
8	49–66	NPEKPHFPLATEDDVRHH
9	56–73	PLATEDDVRHHFTPSERQ
10	63–80	VRHHFTPSERQLCLSSIQ
11	70–87	SERQLCLSSIQTAFNQGA
12	77–94	SSIQTAFNQGAGTCALSD
13	84–101	NQGAGTCALSDSGRISYT
14	91–108	ALSDSGRISYTVEFSLPT
15	98–115	ISYTVEFSLPTQHTVRLI
16	105–123	SLPTQHTVRLIRATASPSA
3m	15–32	***D***GQPVNQLCQMLGKIIAQ
7m	42–59	GKKN***K***KKNPEKPHFPLAT
12m	77–94	SSIQTAFNQGAGTC***T***LSD

### Isolation of porcine peripheral blood mononuclear cells

Peripheral blood from PRRSV-free pigs was collected into heparin-coated collection tubes (Becton Dickinson), diluted 1:1 in RPMI 1640 (GIBCO), overlaid on Ficoll-Hypaque (Amersham Biosciences), and centrifuged at 500 × *g* for 20 min. Peripheral blood mononuclear cells (PBMCs) were washed three times in RPMI 1640, and resuspended in advanced RPMI 1640 medium, which contains 25 mM HEPES (GIBCO), 2 mM L-glutamine (GIBCO), and 100 U/ml penicillin G, 100 mg/ml streptomycin and 0.25 mg/ml amphotericin B (antibiotic/antimycotic solution; GIBCO). All animal protocols were approved by the Animal Care and Ethics Committee of Nanjing Agricultural University (permit number: IACECNAU 20121001) and followed the Guiding Principles for Biomedical Research Involving Animals.

### Generation of porcine MoDCs

Porcine MoDCs were prepared as previously reported [29] with minor modifications. Freshly isolated PBMCs were placed into 75-cm^2^ tissue culture flasks (Corning) and incubated for 3 h in an advanced RPMI 1640 medium at 37°C in 5% CO_2_. Non-adherent cells were removed by washing with RPMI 1640. Adherent cells were cultured in complete RPMI 1640 medium [10% heat-inactivated FBS (GIBCO), 25 mM HEPES (GIBCO), 2 mM L-glutamine (GIBCO), and 100 U/ml penicillin G, 100 mg/ml streptomycin and 0.25 mg/ml amphotericin B (antibiotic/antimycotic solution; GIBCO)] containing 20 ng/ml recombinant porcine granulocyte–macrophage colony-stimulating factor (rpGM-CSF; R&D Systems) and 20 ng/ml recombinant porcine IL-4 (rpIL-4; R&D Systems) at 37°C in 5% CO_2_. Cells were incubated for 5 days with replacement of 50% of medium on day 3. The MoDCs were harvested on day 5 using cell dissociation enzyme-free Hanks’-based buffer (GIBCO) and resuspended in advanced RPMI 1640 medium.

For confirmation of MoDCs generation, the MoDCs were determined for the expression of CD80, CD86, and MHC class II molecules [30], using mouse anti-human CD80-FITC (BD Bioscience), anti-human CD86-PE (BD Bioscience) (they can cross react with the pig CD80 and CD86), and anti-pig MHC-II (IgG1, Abcam), which was follow conjugated with anti-mouse IgG (H+L)-APC (Life Technologies), respectively.

### Construction of recombinant baculoviruses expressing PRRSV structural proteins

Open reading frames (ORFs) 2–7 of HP-PRRSV strain BB0907 were amplified with the special primers (S1 Table) and cloned into the pFastBac Dual vector. The forward primers of each ORF contained a kozac sequence CCACC**ATG**G, and the reverse primers of each ORF contained a 6× His tag and the stop codon. Recombinant baculoviruses were obtained according to the manufacturer’s protocol of the Bac-to-Bac^®^ Baculovirus Expression System (Invitrogen). Protein expression was determined by infecting SF9 cells (Invitrogen) with the recombinant baculoviruses. Recombinant proteins were purified using Ni-NTA resin (Invitrogen), concentrated using ultrafiltration devices (Millipore) and resuspended in phosphate-buffered saline (PBS). The concentrated protein was quantified using a Bradford assay (Bio-Rad), and stored at –20°C until needed.

### Construction of infectious cDNA clones of PRRSV

The full-length genome of HP-PRRSV strain BB0907 was amplified using the five primer pairs listed in S1 Table. A recombinant plasmid (pCMV-BB0907) containing the full-length cDNA of the BB0907 was constructed as shown in S1 Fig. To introduce the amino acid mutations of N protein of S1 into PRRSV infectious cDNA clone pCMV-BB0907, we used site-directed mutagenesis as previously describe [33]. Moreover, a reverse mutant that contained 15N and 46R in N protein was constructed from the full-length infectious cDNA clone pCMV-BN-N15D/R46K using the site-directed mutagenesis (S1 Fig).

### Rescue of recombinant PRRSV

The plasmids carrying the full-length PRRSV cDNA were individually transfected into Marc-145 cells using Lipofectamine 2000 (Invitrogen). Four days after transfection, recombinant viruses were obtained and cloned by plaque assay. The mutations in the rescued viruses were confirmed by RT-PCR and sequencing.

### PRRSV infection and protein stimulation in MoDCs

MoDCs were infected with PRRSV at a multiplicity of infection (m.o.i.) of 0.1 for 1 h at 37°C in advanced RPMI 1640 medium. To eliminate nonabsorbed virus, cells were washed three times at 200 × *g* at 4°C, and resuspended in fresh complete RPMI 1640 medium. The infected MoDCs (2 ×10^5^) were seeded into 24-well tissue culture plates (Corning) and after 24 h, 2 ×10^6^ autologous PBMCs were added to each well for co-culture. Recombinant proteins and synthetic peptides were added into the MoDCs (2×10^5^) that were cultured in a 24-well plate with a final concentration of 5 μg/ml, and after 24 h, 2 ×10^6^ autologous PBMCs were added. The lysates of mock-infected Marc-145 cells or PBS were used as controls.

### Flow cytometry

Tregs were determined by coexpression of CD4, CD25 and Foxp3. After 3 days co-culture with PBMCs and MoDCs [34], all cells were harvested and stained with anti-porcine CD25 (AbD Serotec), followed by APC goat anti-mouse IgG (H+L) (Life Technologies) and goat anti-porcine CD4a-FITC (Clone 74-12-4; SouthernBiotech). Foxp3 intracellular staining was performed with anti-rat/mouse Foxp3-PE (Clone FJK-16s; eBioscience), which had cross-reactivity with swine Foxp3 [26], using the Foxp3 Staining Buffer Set (Staining, Fixation/Permeabilization and Permeabilization Buffers; eBioscience), to obtain the three-color staining CD4^FITC^ CD25^APC^ Foxp3^PE^. The Tregs frequency was evaluated by flow cytometry (BD FACS Canto II), and data were analyzed using FlowJo version 7.6.1 software.

### Sorting CD4^+^ CD25^high^ cells

After 3 days co-culture with PBMCs and PRRSV or Marc-145 lysate-treated MoDCs, all cells were harvested and stained with an appropriate concentration of anti-CD4-FITC and anti-CD25-APC as described previously. Enrichment of CD4^+^ CD25^high^ lymphocytes was sorted on a FACSAria cell sorter (BD Biosciences).

### ELISA

The supernatants from 3 days co-culture were collected and the levels of secreted IL-10 and TGF-β were quantified using commercial ELISA kits (R&D Systems).

### Suppressive activity assay

The suppressive activity of PRRSV-induced Tregs was evaluated by determining the proliferation of phytohemagglutinin (PHA)-treated, autologous PBMCs cultured with the CD4^+^ CD25^high^ lymphocytes that sorted from lymphocytes exposed to the PRRSV-infected MoDCs. Because of PRRSV cultures with Marc-145 cells, the lymphocytes exposed to Marc-145 lysate-treated MoDCs were included in the experiment as negative controls [29, 30]. In brief, autologous PBMCs (~10^7^) resuspended in 1 ml PBS/0.1% bovine serum albumin were labeled with CSFE (Invitrogen) for 10 min at 37°C, with a final working concentration of 10 μM. Following incubation, 0.5 ml FBS (GIBCO) was added to stop the staining reaction. CSFE-labeled PBMCs (~10^5^ cells/well, resuspended in complete medium) were co-cultured with the sorted CD4^+^ CD25^high^ lymphocytes in a 96-well plate at a Treg:PBMC ratios of 1:10, 2.5:10 and 5:10, in the presence of 5 mg/ml PHA (Sigma–Aldrich) for 5 days at 37°C in a 5% CO_2_ incubator. The results are expressed as percentage of suppression determined with the formula: % suppression = 100 × [1 − (% proliferation w/PRRSV/ % proliferation w/mock)] [35].

### Statistical analysis

Data were analyzed using paired Student’s one-way analysis of variance (ANOVA). Differences among treatments were determined by Tukey’s test (*P*<0.05). Analyses were performed with PRISM version 5.02 software (GraphPad).

## Results

### Induction of Tregs from PBMCs co-cultured with HP-PRRSV-infected MoDCs

**Fig 1:**
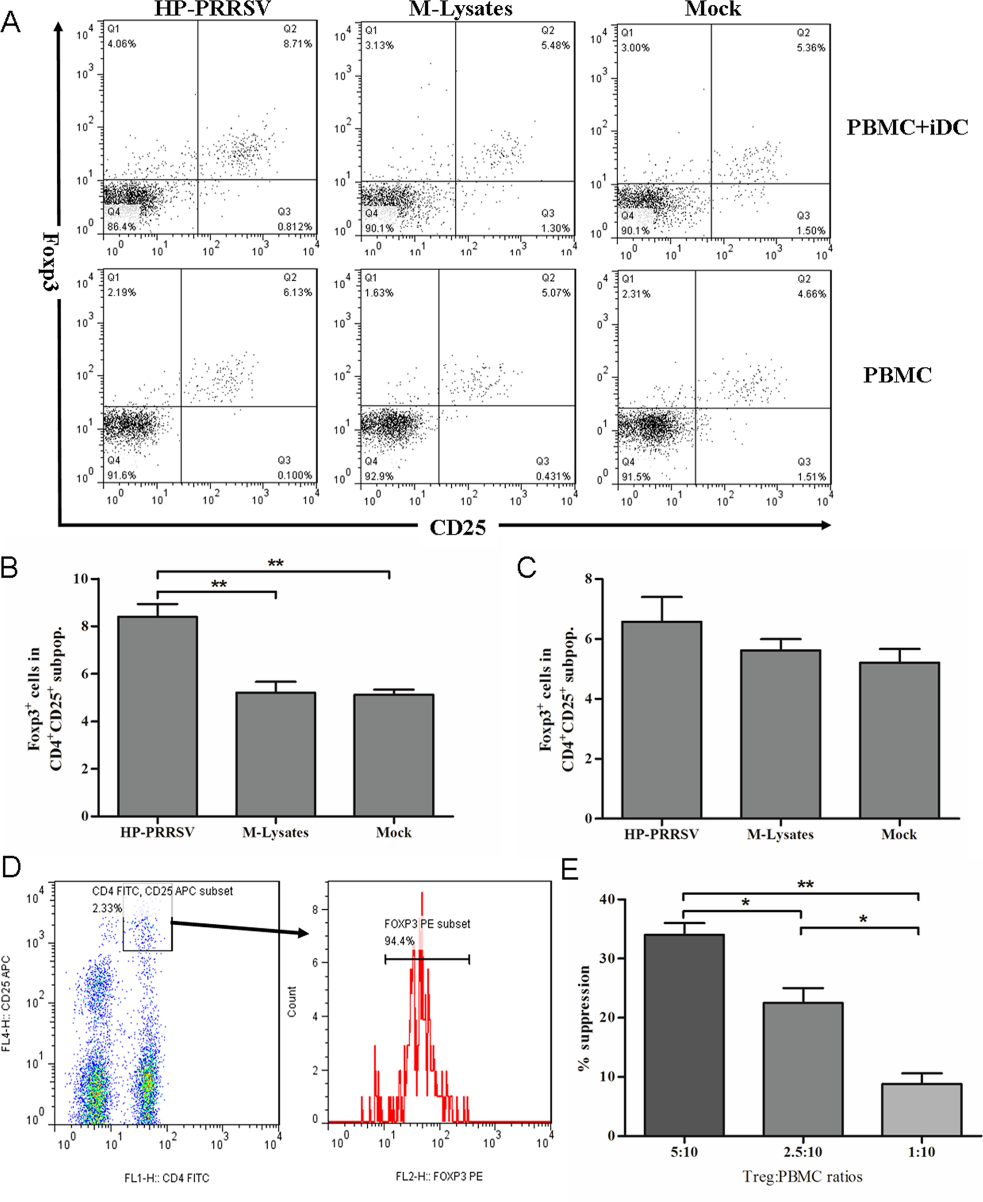
Induction of Tregs from lymphocytes co-cultured with PRRSV-infected MoDCs. (A) Representative flow cytometry profile of lymphocytes following 3 days co-culture of PBMCs with PRRSV-infected MoDCs (upper layer), and only culture with PRRSV-infected PBMCs (lower layer). (B) Percentage of Foxp3^+^ cells in the gated CD4^+^CD25^+^ subpopulations of PBMCs co-cultured with PRRSV-infected MoDC. (C) Percentage of Foxp3^+^ cells in the gated CD4^+^ CD25^+^ subpopulations of PRRSV-infected PBMCs alone. (D) Sorted CD4^+^CD25^high^ cells. (E) Percentage suppression at the indicated Tregs: peripheral lymphocyte ratios. The lymphocytes exposed to Marc-145 lysate-treated MoDCs were used as negative control. The percentage of suppression was calculated as follows: % suppression = 100 × [1 − (% proliferation w/PRRSV/ % proliferation w/mock)] [35]. Data from three independent experiments. All data analysis was done using one-way ANOVA and significant differences are shown (*P<0.05 and **P< 0.01).

To evaluate the role of HP-PRRSV in induction of Tregs, porcine MoDCs were generated from PBMCs using rpGM-CSF and rpIL-4. The porcine MoDCs exhibited significant enhanced expressions of CD80, CD86 and MHC class II molecules on the cellular surface, compared with the freshly isolated lymphocytes (S2 Fig). This result indicated that the MoDCs were generated successfully. In addition, the HP-PRRSV strain could successfully infect MoDCs as previous reports (S3 Fig) [33, 36]. As shown in Fig 1, The CD4^+^ CD25^+^ Foxp3^+^ expression was determined on autologous PBMCs co-cultured with HP-PRRSV-infected MoDCs using flow cytometry. The percentage of CD4^+^CD25^+^Foxp3^+^ Tregs among HP-PRRSV-infected MoDCs was significantly higher than that co-cultured with mock-infected MoDCs (*P*<0.01) (Fig 1A and 1B). The percentage of Tregs was not significantly increased in HP-PRRSV-infected PBMCs alone, compared with that treated with Marc-145 cell lysates (M-Lysates) (*P*>0.05) (Fig 1A and 1C).

The CD4^+^CD25^+^Foxp3^+^ lymphocytes were only observed in the CD4^+^CD25^high^ subpopulation [30]. Suppressive function was the hallmark for the definition of Tregs, therefore, the suppressive activity of the PRRSV-induced Tregs was determined by an *in vitro* suppression assay. In order to obtain the alive Tregs, we sorted the CD4^+^CD25^high^ subpopulation and analyzed the expression of Foxp3. As shown in Fig 1D, the sorted CD4^+^CD25^high^ cells were almost all Foxp3^+^ Treg cells (>94.0%). Compared to mock treatment, the PRRSV-induced Tregs had a significant suppressive effect on proliferation of PHA-stimulated lymphocytes. The level of suppression was correlated with the number of Tregs that was added to the culture system (Fig 1E).

### Effects of different PRRSV strains on Tregs induction

**Fig 2:**
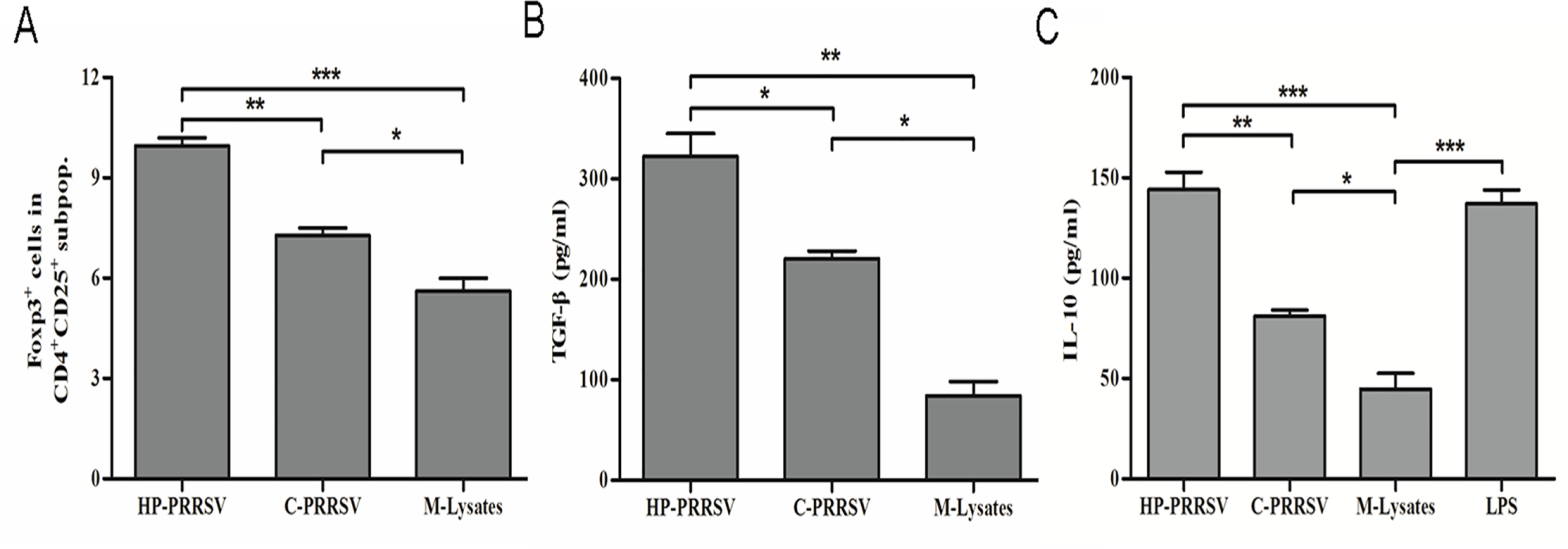
Effects of different PRRSV strains on Tregs induction. (A) Percentage of Foxp3^+^ cells in the gated CD4^+^ CD25^+^ subpopulations of HP-PRRSV and C-PRRSV. (B) Concentrations of TGF-β in the supernatants of 3-day co-cultures. (C) Concentrations of IL-10 in supernatants of 3-day co-cultures. Data came from three independent experiments. Data analysis was done using one-way ANOVA and significant differences are shown (*P<0.05, **P< 0.01 and ***P<0.001).

Previous studies have demonstrated that type 2 PRRSV induce Tregs proliferation [29, 30, 37], but type 1 strains do not [32]. We compared Tregs inductions by HP-PPRSV strain BB0907 and C-PRRSV strain S1 in the co-culture system. The percentage of Tregs (CD4^+^CD25^+^Foxp3^+^) was significantly increased in HP-PRRSV and C-PRRSV when compared to the M-Lysates control group (*P*<0.05). However, HP-PRRSV had greater proliferation of Tregs than C-PRRSV had (Fig 2A). Both TGF-β and IL-10 were significantly increased by HP-PRRSV BB0907 and C-PRRSV S1 compared with in the M-Lysates control group. The levels of TGF-β and IL-10 in HP-PRRSV BB0907 group were significantly higher than those in the C-PRRSV S1 group (*P*<0.05) (Fig 2B and 2C).

### Role of N protein of HP-PRRSV in Tregs induction

**Fig 3:**
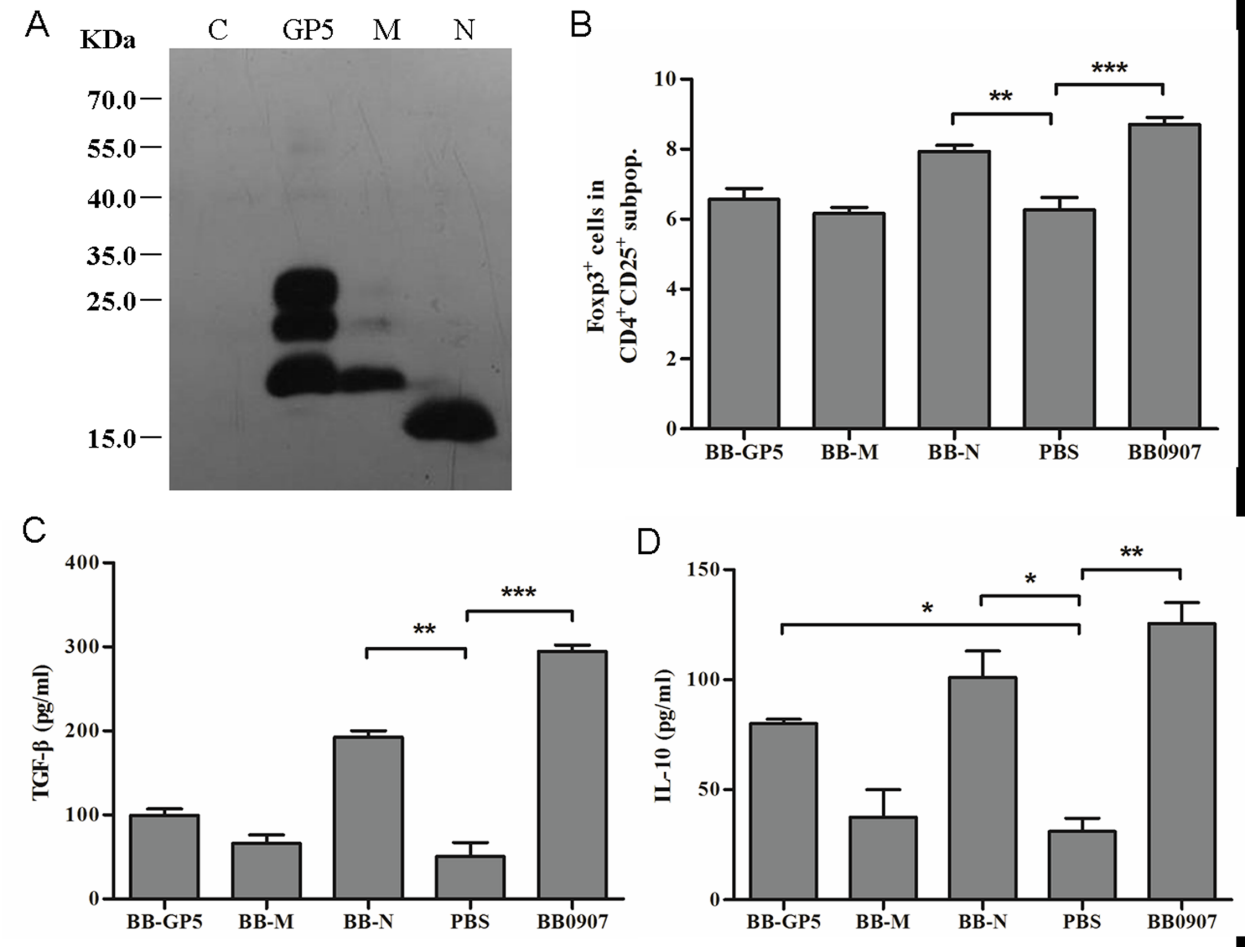
Role of baculovirus-expressed proteins GP5, M and N of HP-PRRSV in induction of Tregs proliferation. (A) Purified recombinant GP5, M and N protein expressed in baculovirus. (B) Percentage of Foxp3^+^ cells in the gated CD4^+^ CD25^+^ subpopulations of GP5, M and N. (C) Concentrations of TGF-β in supernatants of 3-day co-cultures. (D) Concentrations of IL-10 in supernatants of 3-day co-cultures. Data came from three independent experiments. Data analysis was done using one-way ANOVA and significant differences are shown (*P<0.05, **P<0.01 and ***P<0.001).

To examine the effect of PRRSV structural proteins on induction of Tregs, the GP5, M and N proteins of HP-PRRSV were expressed in baculovirus expression systems and confirmed by western blotting with His antibody (Fig 3A). Recombinant GP5, M and N proteins were purified and used to examine their effects on Tregs induction. Only N protein significantly induced proliferation of Tregs (Fig 3B). The concentrations of TGF-β and IL-10 in the supernatants were quantified with ELISA. GP5 and N proteins significantly increased expression of IL-10 (*P*<0.05), but only N protein significantly increased TGF-β expression (*P*<0.05) (Fig 3C and 3D).

**Fig 4:**
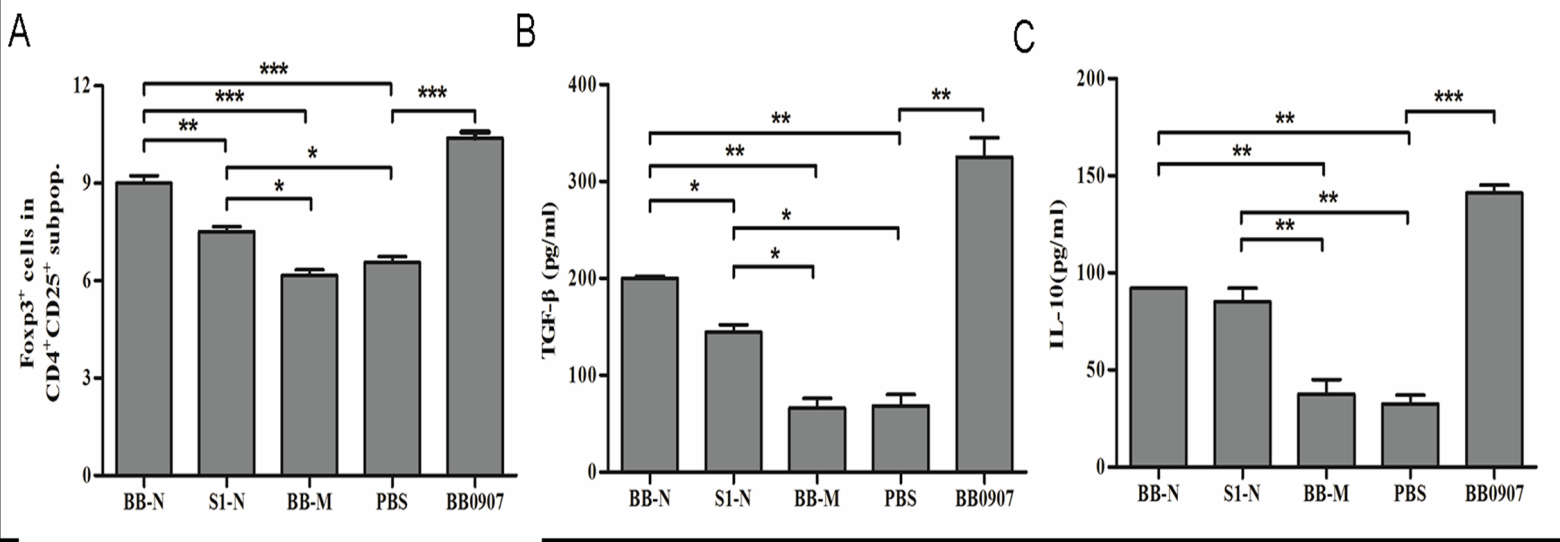
Effects of N protein of different virulent PRRSV strains on Tregs induction. (A) Percentage of Foxp3^+^ cells in the gated CD4^+^CD25^+^ subpopulations of BB-N and S1-N. The BB-M was negative control. (B) Concentrations of TGF-β in supernatants of 3-day co-cultures. (C) Concentrations of IL-10 in supernatants of 3-day co-cultures. Data from three independent experiments. Data analysis was done using one-way ANOVA and significant differences are shown (*P<0.05, **P<0.01 and ***P<0.001).

The effects of N protein from different PRRSV on Treg induction were determined. HP-PRRSV BB0907 and C-PRRSV S1 N proteins (BB-N and S1-N) significantly induced proliferation of Tregs (*P*<0.05) (Fig 4). The percentage of Tregs in the BB-N group was significantly higher than in the S1-N group (*P*<0.05) (Fig 4A). BB-N and S1-N significantly increased expression of TGF-β and IL-10, compared to the M-Lysates control group (*P*>0.05) (Fig 4B and 4C). The levels of TGF-β in the BB-N group were significantly higher than that in the S1-N group (*P*<0.05) (Fig 4B).

### Key amino acids residues of NP involved in Tregs induction

**Fig 5:**
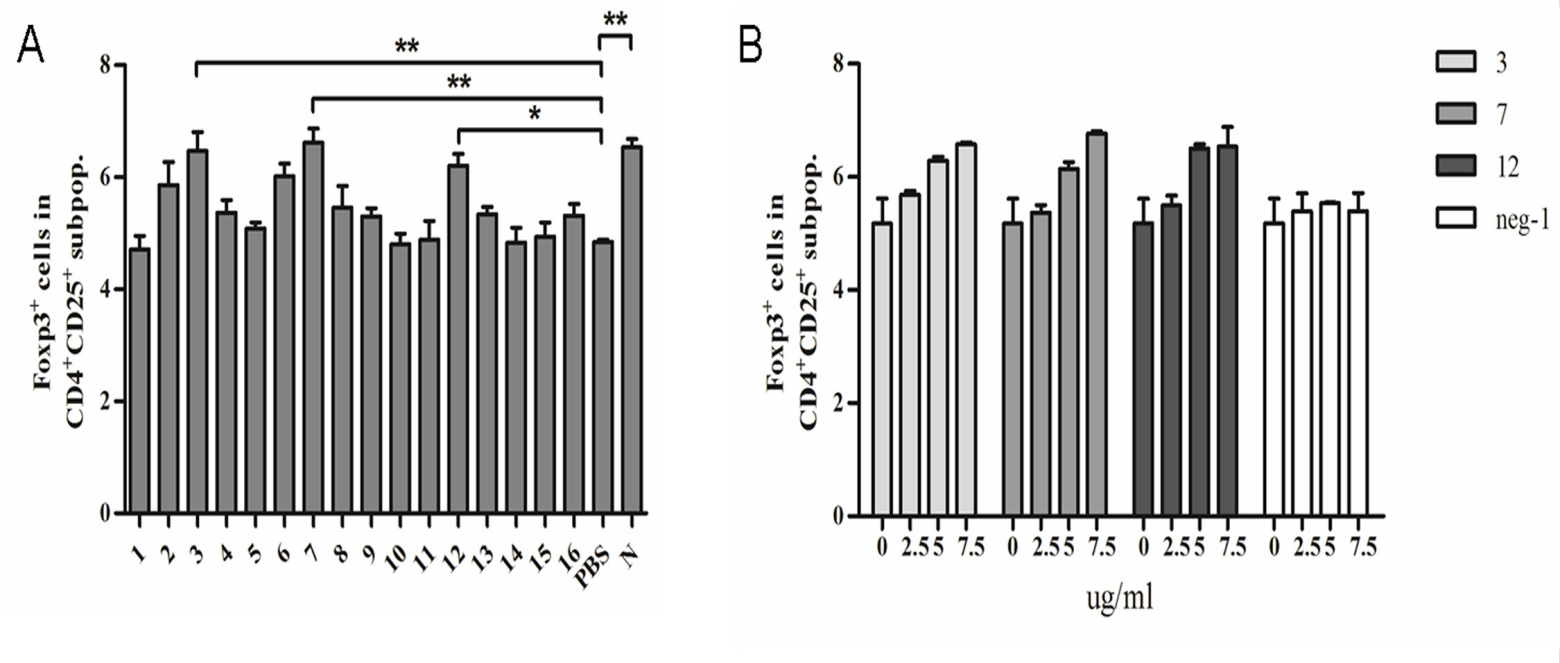
Effects on Tregs induction of synthetic peptides of N protein of HP-PRRSV. (A) Percentage of Foxp3^+^ cells in the gated CD4^+^CD25^+^ subpopulations of 16 synthetic peptides, which covered the whole length of HP-PRRSV N protein. (B) The dose-dependent manner of three peptides 3, 7 and 12. Data came from three independent experiments. Data analysis was done using one-way ANOVA and significant differences are shown (*P<0.05 and **P< 0.01).

To define the regions of NP of HP-PRRSV involved in induction of Tregs, 16 peptides, which covered the whole length of N protein, were synthesized (Table 1) and used to induce Tregs in the co-culture system. Peptides 3 (aa 15–21), 7 (aa 42–48) and 12 (aa 88–94) significantly induced Tregs compared to PBS (Fig 5A). All three peptides induced Tregs in a dose-dependent manner (Fig 5B).

**Fig 6:**
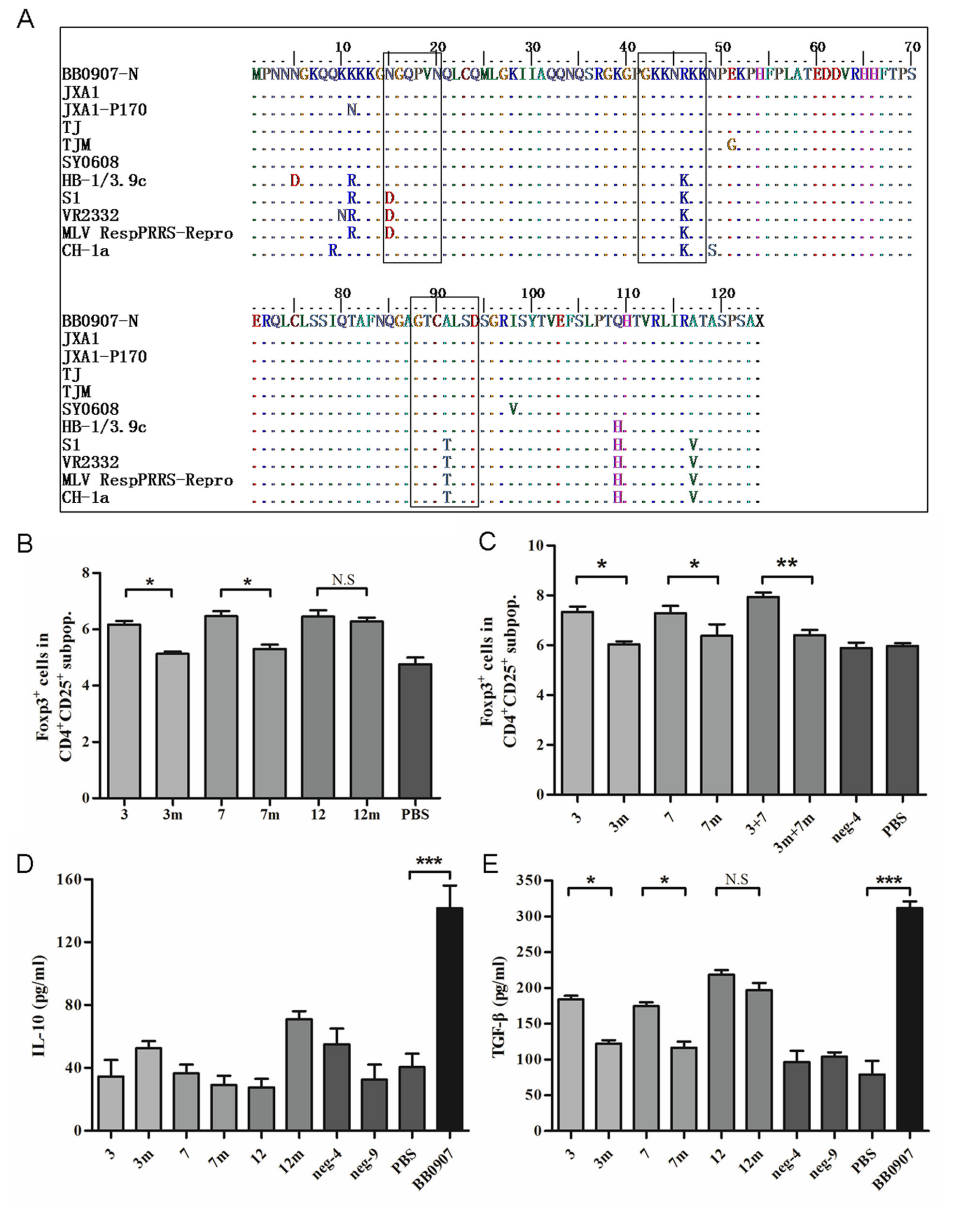
15N and 46R in HP-PRRSV N protein were key amino acids in Tregs induction. (A) Alignment of amino acid sequences of N protein of PRRSV BB0907, S1 and other representative strains. (B) Percentage of Foxp3^+^ cells in the gated CD4^+^CD25^+^ subpopulations of three pairs of synthetic peptides. (C) Additivity assay of 3 and 7 peptides in Treg induction. (D) Concentrations of IL-10 in supernatants of 3-day co-cultures. (E) Concentrations of TGF-β in supernatants of 3-day co-cultures. Data came from three independent experiments. Data analysis was done using one-way ANOVA and significant differences are shown (*P<0.05, **P<0.01 and ***P<0.001).

As a result of the different levels of induction of Tregs proliferation by the N proteins of HP- and C-PRRSV, the N protein sequences of reference strains were compared. C-PRRSV NP had three different substitutions at aa 15–21 (N15D), 42–48 (R46K) and 88–94 (A91T), compared to those of BB-N (Fig 6A). Thus, three mutant peptides, 3m, 7m and 12m, were synthesized according to these substitutions (Table 1), and were used to induce Tregs in the co-culture system. The levels of Tregs in the 3m and 7m groups were significantly lower than those in peptides 3 and 7, respectively (Fig 6B). The effects of Tregs induction could be enhanced by adding peptides 3 and 7 together (Fig 6C).

The concentrations of IL-10 and TGF-β in the supernatants were measured. The production of IL-10 did not obviously differ according to peptide treatment (Fig 6D). However, TGF-β production significantly decreased with peptides 3m and 7m, compared to that with peptides 3 and 7 (*P*<0.05) (Fig 6E).

These results indicate that 15N and 46R in BB-N might be the key amino acid residues involved in inducing Tregs proliferation.

### Recovery of PRRSV harboring mutant in N-protein

**Fig 7:**
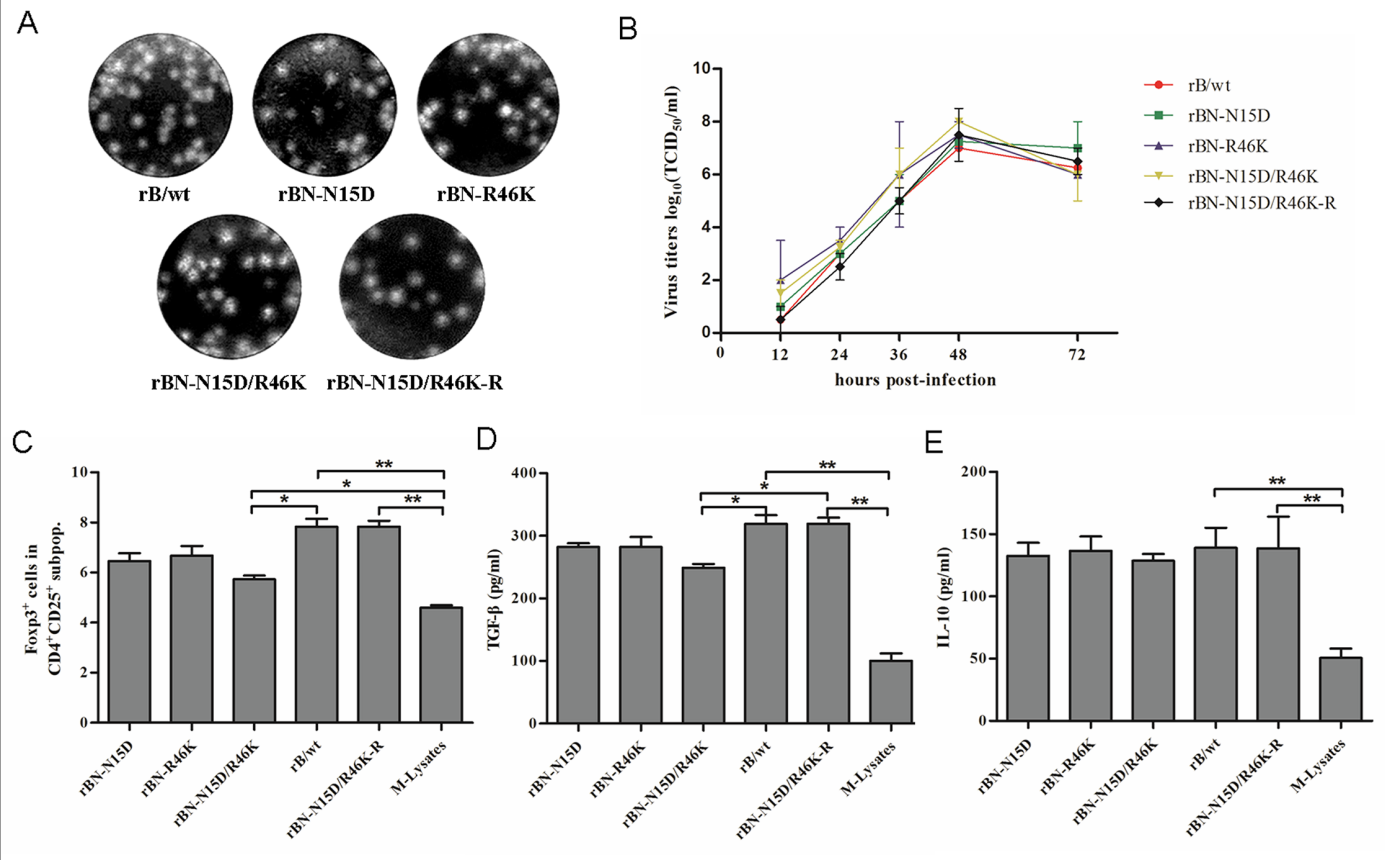
Effects of the mutant recombinant PRRSV strains on Tregs induction. (A) Plaque morphology assay of the recombinant mutant strains. (B) Growth kinetics of the recombinant mutant strains. (C) Percentage of Foxp3^+^ cells in the gated CD4^+^CD25^+^ subpopulations of the recombinant viruses. (D) Concentrations of TGF-β in the supernatants of 3-day co-cultures. (E) Concentrations of IL-10 in the supernatants of 3-day co-cultures. All data are represented as the means ± standard deviation of three independent experiments, and data analysis was done using one-way ANOVA and significant differences are shown (*P<0.05 and **P<0.01).

To confirm the role of the N protein residues described above in induction of Tregs proliferation, a standard reverse genetics approach was used to recover these mutant viruses (S1 Fig). Three recombinant PRRSVs, rBN-N15D [asparagine (N) at residue 15 mutated to aspartic acid (D)], rBN-R46K [arginine (R) at residue 46 mutated to lysine (K)] and rBN-N15D/R46K (residues 15 and 46 mutated to D and K), containing the mutations N15D, R46K and N15D/R46K in the N protein, were rescued. To confirm that the results were due to the amino acid mutants, a revertant virus rBN-N15D/R46K-R was also rescued. They induced plaques in Marc-145 cells, similar to rB/wt (Fig 7A). Multistep growth kinetics of viruses showed that growth of rBN-N15D, rBN-R46K and rBN-N15D/R46K and rBN-N15D/R46K-R was similar to that of rB/wt (*P*<0.05) (Fig 7B).

### Regulation of recombinant PRRSV on Tregs

To investigate the role of rBN-N15D, rBN-R46K and rBN-N15D/R46K in regulation of Tregs, MoDCs were infected with rB/wt, rBN-N15D, rBN-R46K and rBN-N15D/R46K at an m.o.i. of 0.1. CD4^+^CD25^+^Foxp3^+^ expression was determined on PBMCs co-cultured with PRRSV-infected MoDCs using flow cytometry. rBN-N15D/R46K significantly reduced Tregs proliferation when compared with rB/wt (*P*<0.05) and the number of Tregs induced by rBN-N15D and rBN-R46K was lower than that with rB/wt (*P*>0.05) (Fig 7C). IL-10 production by rBN-N15D, rBN-R46K and rBN-N15D/R46K did not differ significantly from that of the parental virus rB/wt. rBN-N15D/R46K significantly reduced production of TGF-β compared to rB/wt (*P*<0.05) (Fig 7D and 7E). The revertant virus rBN-N15D/R46K-R caused similar Tregs induction and production of TGF-β and IL-10 as rB/wt.

## Discussion

PRRS is a pandemic disease that especially affects neonates within the “critical window” of immunological development. PRRSV suppresses innate immunity, causes abnormal B cell proliferation and repertoire development, disrupts normal T cell development in the thymus, and impairs cytotoxic T lymphocyte induction [38]. PRRSV infection can induce CD4^+^CD25^+^Foxp3^+^ Tregs [29, 30, 37], which play an important role in suppressing the immune and inflammatory responses. However, the Tregs epitopes that are crucial for the interactions between PRRSV and the host immune system have not been explored. In this study, our results showed that Tregs induction by HP-PRRSV was higher than that by C-PRRSV in MoDCs. The aa 15–21, 42–48 and 88–94 in HP-PRRSV N protein were important for inducing Tregs, using 16 synthetic peptides that covered the whole length of N protein. By using mutant synthetic peptides and reverse genetic methods, it was firstly found that the two key residues, 15N and 46R, in N protein were involved in inducing of Tregs proliferation. The phenotype of induced porcine Tregs closely resembled that of TGF-β-secreting Th3 Tregs in humans, mice and swine.

The ability to induce a rapid interferon (IFN) response is important for viral clearance and heterologous protection by vaccination [39, 40]. However, infection and vaccination with PRRSV induces a rapid, non-neutralizing antibody response and an early, weak, nonspecific IFN response [41, 42]. The inadequate IFN response may be due to the ability of PRRSV to stimulate Tregs *in vitro* [43]. PRRSV N protein downregulated IFN-β mRNA level in polyI:C-treated immortalized porcine alveolar macrophages by interfering with double-stranded-RNA-induced phosphorylation and nuclear translocation of IFN-regulatory factor 3 [44]. It revealed that N protein is related to suppression of the innate immune response. We showed that HP-PRRSV N protein had greater Tregs induction than C-PRRSV had. A previous study has suggested that type 2 PRRSV N protein induces IL-10, but Tregs induction is not strong [31]. MoDCs infected with type 1 PRRSV strains produce neither TGF-β nor Tregs [32]. These differences may be due to the different strains and the sources of recombinant N protein that expressed with the baculovirus or *Escherichia coli* expression systems.

The N protein of PRRSV has five important antigenic regions at aa 30–52, 37–52, 52–69, 69–112, and 112–123 [45, 46]. The aa 112–123 at the C terminus of the N protein are crucial to maintain structural conformation [47]. A cryptic nuclear localization signal (NLS), called NLS-1, a functional NLS (NLS-2), and a nucleolar localization sequence (NoLS) are, respectively, located at aa 10–13, 41–47, and 41–72, respectively [48]. The dominant Tregs epitopes that are located in the core and NS3 proteins of HCV have been determined by using peptide pools, which suggest that stimulation with single short foreign peptide is sufficient to cause rapid and dramatic induction of Tregs in culture [49, 50]. To screen the key amino acids that are related to induction of Tregs, 16 synthetic peptides that covered the whole length of N protein and mutants of three amino acid regions were used to stimulate MoDCs. The results showed that peptides 3 (aa 15–21), 7 (aa 42–48) and 12 (aa 88–94) significantly induced Tregs proliferations compared to PBS. Three mutant peptides, 3m, 7m and 12m, were synthesized according to these substitutions (15–21 (N15D), 42–48 (R46K) and 88–94 (A91T)). And the levels of Tregs in the 3m and 7m groups were significantly lower than those in peptides 3 and 7, respectively. The results of the structures of the peptides between HP-PRRSV and C-PRRSV analyzed by using SPLIT 4.0 software revealed that the indexes of α-helix and β-fold of 3m were higher than peptide 3. And the index of α-helix of 7m has a litter rise than that of peptide 7 (data not shown here). The amino acid 15N is close to NLS-1 and 46R is located in the NLS-2 region, which interacts with the nuclear transporters importin α and β [48]. A knockout mutant for the NLS-2 in the PRRSV N protein was engineered using reverse genetics. Pigs infected with this mutant developed reduced viremia and significantly higher neutralizing antibody titers [51]. This suggests that the NLS-2 region has an important immunomodulatory domain. The real structures of the peptides and the relationship between protein structure and Tregs induction should be studied in the future.

Cell-mediated immune responses, including CD4^+^, CD8^+^ and CD4^+^/CD8^+^ double-positive T cells, have been detected in PRRSV-infected animals, and they appear transiently from 2 to 8 weeks post-infection [18, 52]. The GP5 protein is associated within the virion to the membrane protein M via disulfide bonds [53]. Both these major envelope proteins induce a robust cellular immune response in PRRSV-exposed pigs [54–56]. They also produce neutralizing antibodies against GP5 and M [57, 58]. In this study, we constructed a total of six recombinant baculoviruses that expressed GP2, GP3, GP4, GP5, M and N proteins of HP-PRRSV. But only GP5, M and N proteins were expressed at high concentrations. The possible reasons why GP2, GP3 and GP4 were not expressed might be related to the high hydrophobicity and structures of these proteins, the expression efficiency of the Bac-to-Bac^®^ baculovirus expression system or others we do not know. We also noted that three bands were observed in the expressed GP5. This expression form was similar to the results obtained by Hyun et al in 2008, and might be related with the levels of glycosylation in insect cells [59]. However, the recombinant GP5 and M proteins were unable to induce Tregs proliferation. And the role of proteins GP2, GP3 and GP4 in inducing Tregs proliferation should be studied in the future.

PRRSV nonstructural proteins (NSPs) can negatively modulate host innate immunity. Nsp1α, nsp1β, nsp2 and nsp11 are antagonists of IFN induction, with different molecular mechanisms [60]. Suppression of innate immunity can be an important contributing factor to the modulation of host immune responses, because type I IFN promotes antigen presentation and natural killer cell function, enhances antibody production of B cells, and plays an important role in differentiation of CD4^+^ and CD8^+^ T cells. Considering the immune dysregulation of NSPs, whether HP-PRRSV NSPs, especially the nsp1, nsp2 and nsp11, have the ability of Tregs induction can be investigated in the future.

DCs play an important role in initiating immunity and maintaining self tolerance. DC subsets specifically stimulate activation and differentiation of Tregs [61–63]. Induction and maintenance of Tregs *in vivo* is dependent on a variety of factors that include co-stimulatory molecules such as CD80 and CD86 [62, 64] and cytokines such as TGF-β [65] and IL-2 [66]. The CD80/86 co-stimulatory molecule on antigen-presenting cells is required for delivering a second signal necessary for activation of T cells [67]. Type 1 and 2 PRRSV have an up- or downregulatory effect on expression of CD80/86 [16, 36, 68–70]. Differences between PRRSV strains in regulation of these molecules have been observed *in vitro* [69, 71]. In the present study, Treg induction by different virulent PRRSV strains might be dependent on the different regulatory effect on modulation of cell-surface co-stimulatory molecules CD80 and CD86. The mechanisms contributing to Tregs induction should be further studied. By the way, the time of co-culture of lymphocytes with virus-infected MoDCs was 3 days in this study, which was the same as T. E. Cecere described [34], but different from that times as previously describe [29], because the morphologic evidence of cytolysis of MoDCs began approximately 5 days post-inoculation with HP-PRRSV strain. It might be related to the cytopathic characteristic of the PRRSV strain on DCs. But the results showed that the numbers of Tregs induced by PRRSV or viral proteins were increased significantly compared with those in control group on the condition of 3 days co-culture.

In summary, in this study, it was firstly found that HP-PRRSV N protein induces Tregs *in vitro* and the dominant epitopes are located at 15N and 46R in N protein. These results data should be helpful for understanding the immune mechanism of PRRSV and developing new vaccines in the future.

## Supporting Information

S1 Fig Construction strategy for infectious cDNA clones of the recombinant PRRSV using BB0907 strain. Full genome of BB0907 was divided into fragments A–D, which were continuously assembled using the restriction enzymes to obtain the full-length clone, pCMV-BB0907. Fragment D encoding the structural proteins was amplified and cloned into the pEASY-Simple Blunt vector using *Asc*I and *Spe*I restriction endonucleases, yielding pEASY-B-D, which was used as the intermediate plasmid. The site-directed mutants were constructed using pEASY-BB-D as the template, and fragment D of pCMV-BB0907 was replaced by the analogous fragments derived from mutants of pEASY-BB-D, which resulted in the generation of full-length mutant clones.
(TIF)

S2 Fig Expression analyses of CD80, CD86 and MHC II molecules on the cellular surface of MoDCs and freshly isolated PBMCs. The white histograms represent the PBMCs, and the grey histograms represent the cultured MoDCs.
(TIF)

S3 Fig Infection analysis of MoDCs by HP-PRRSV strain with IFA. The MoDCs cultured in 24-well tissue culture plates (2×10^5^ /well) were infected with HP-PRRSV at m.o.i. of 0.1. After incubation for 1 h at 37°C, the cells were washed with PBS, and incubated in fresh complete RPMI 1640 medium. After 24 h, the cells were rinsed with PBS, fixed with 3.7% formaldehyde, and incubated with mAb against PRRSV N protein (made in our laboratory) and FITC-Goat Anti-mouse IgG for immunofluorescence microscopy. Meanwhile, the non-infected MoDCs were used as negative control. (A) The HP-PRRSV infected MoDCs. (B) The non-infected MoDCs.
(TIF)

S1 Table Primer sequences for construction of the baculovirus structural proteins, the subgenomic replicon of PRRSV, and site-directed mutagenesis. (DOC)
