# Supplementary figures and images for: The 15N and 46R Residues of Highly Pathogenic Porcine Reproductive and Respiratory Syndrome Virus Nucleocapsid Protein Enhance Regulatory T Lymphocytes Proliferation
Source: PLoS One. 2015 Sep 23;10(9):e0138772. doi: 10.1371/journal.pone.0138772 (PMC4580451; doi:10.1371/journal.pone.0138772)

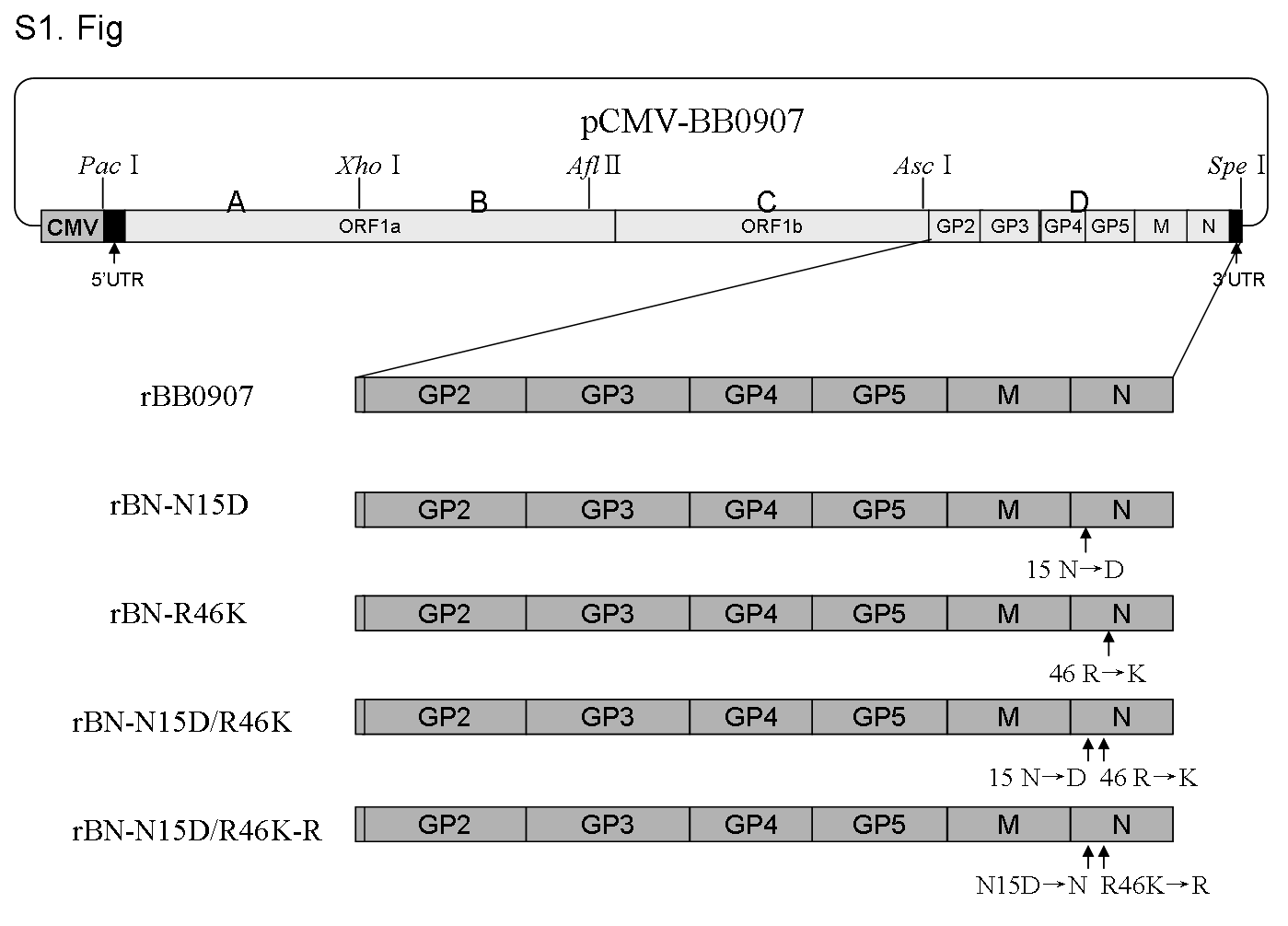

Supplement: S1 Fig — Full genome of BB0907 was divided into fragments A–D, which were continuously assembled using the restriction enzymes to obtain the full-length clone, pCMV-BB0907. Fragment D encoding the structural proteins was amplified and cloned into the pEASY-Simple Blunt vector using AscI and SpeI restriction endonucleases, yielding pEASY-B-D, which was used as the intermediate plasmid. The site-directed mutants were constructed using pEASY-BB-D as the template, and fragment D of pCMV-BB0907 was replaced by the analogous fragments derived from mutants of pEASY-BB-D, which resulted in the generation of full-length mutant clones. (TIF) [file pone.0138772.s001.tif]

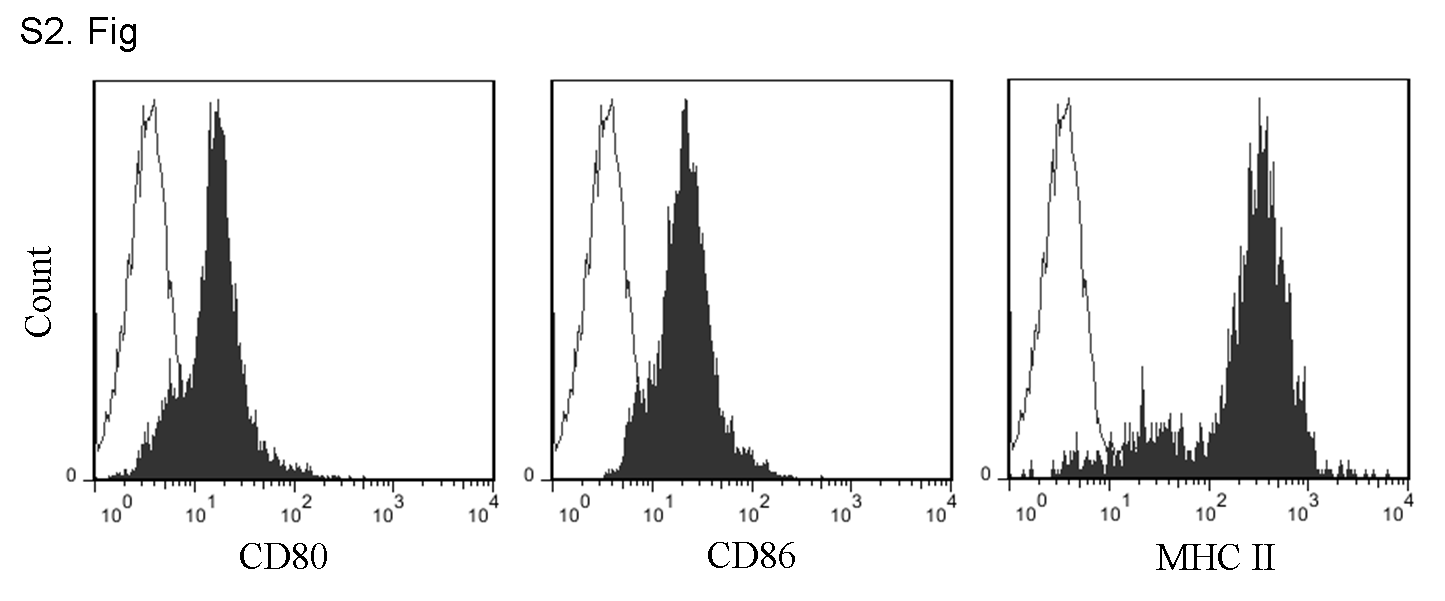

Supplement: S2 Fig — The white histograms represent the PBMCs, and the grey histograms represent the cultured MoDCs. (TIF) [file pone.0138772.s002.tif]

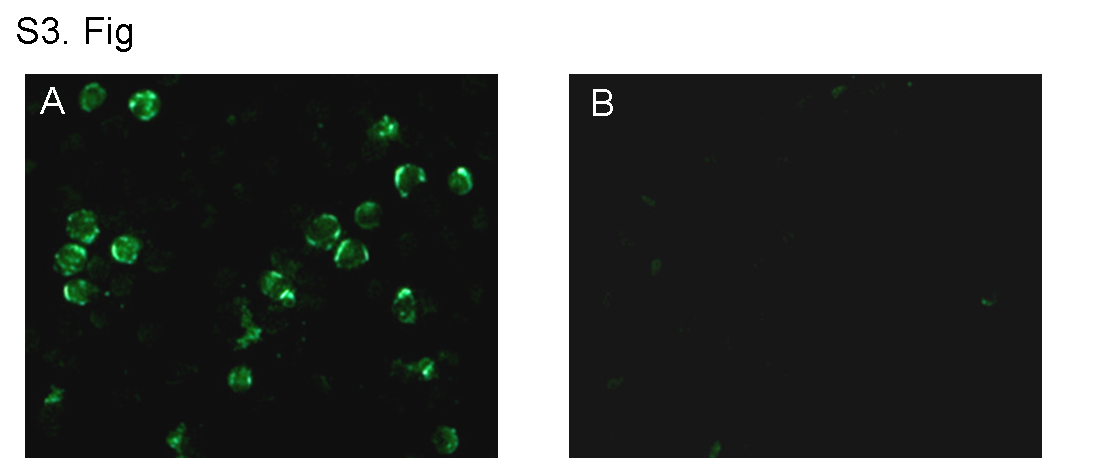

Supplement: S3 Fig — The MoDCs cultured in 24-well tissue culture plates (2×10^5^ /well) were infected with HP-PRRSV at m.o.i. of 0.1. After incubation for 1 h at 37°C, the cells were washed with PBS, and incubated in fresh complete RPMI 1640 medium. After 24 h, the cells were rinsed with PBS, fixed with 3.7% formaldehyde, and incubated with mAb against PRRSV N protein (made in our laboratory) and FITC-Goat Anti-mouse IgG for immunofluorescence microscopy. Meanwhile, the non-infected MoDCs were used as negative control. (A) The HP-PRRSV infected MoDCs. (B) The non-infected MoDCs. (TIF) [file pone.0138772.s003.tif]
