# Supplementary material for: The 15N and 46R Residues of Highly Pathogenic Porcine Reproductive and Respiratory Syndrome Virus Nucleocapsid Protein Enhance Regulatory T Lymphocytes Proliferation
Source: PLoS One. 2015 Sep 23;10(9):e0138772. doi: 10.1371/journal.pone.0138772 (PMC4580451; doi:10.1371/journal.pone.0138772)
Supplement: S1 Table — (DOC) [file pone.0138772.s004.doc]

**S1 Table.** Primer sequences for construction of the baculovirus structural proteins, the subgenomic replicon of PRRSV, and site-directed mutagenesis.

| Primers | Sequence (5’-3’) | Purpose |
| --- | --- | --- |
| Primers for the construction of the baculoviruses of structure proteins of BB0907 | |  |
| p10-BB-GP2-F | ATACTCGAG*CCACCATGG*AATGGGGTCTATGC | Bac-BB/GP2 |
| p10-BB-GP2-R-His | ATAGCATGCTCAGTGATGGTGATGGTGATGCCATGAGTTCAAAAGAAAAG |
| p10-BB-GP3-F | ATACTCGAG*CCACCATGG*CTAATAGCTGTACAT | Bac-BB/GP3 |
| p10-BB-GP3-R-His | ATAGCATGCCTAGTGATGGTGATGGTGATGTCGCCGTGCGGCACTGAG |
| p10-BB-GP4-F | ATACTCGAG*CCACCATGG*CTGCGCCCTTTCTT | Bac-BB/GP4 |
| p10-BB-GP4-R-His | ATAGCATGCTCAGTGATGGTGATGGTGATGAATTGCCAGTAGGATGGC |
| p10-BB-GP5-F | ATACTCGAG*CCACCATGG*TGGGGAAGTGCTTGAC | Bac-BB/GP5 |
| p10-BB-GP5-R-His | ATAGCATGCCTAGTGATGGTGATGGTGATGGAGACGACCCCATTGTTC |
| p10-BB-M-F | ATACTCGAG*CCACCATGG*GGTCGTCTCTAGAC | Bac-BB/M |
| p10-BB-M-R-His | ATAGCATGCTCAGTGATGGTGATGGTGATGTTTGGCATATTTAACAAGG |
| p10-BB-N-F | GCGCTCGAG*CCACCATGG*CAAATAATAACGGC | Bac-BB/N |
| p10-BB-N-R-His | ATAGCATGCTCAGTGATGGTGATGGTGATGTGCTGAGGGTGATGCTGT |
| Primers for the construction of infectious, full-length cDNA clones of BF10 | |  |
| A-1-F | GCGTTAATTAAACCGTCATGACGTATAGGTGTTG | Fragment A |
| A-3408-R | TGTCTCGAGAATCATCTTTGGGAGAAACC | Fragment A |
| B-3383-F | TTCTTAATTAAATGATTCTCGAGACACCGCC | Fragment B |
| B-6473-R | GTGCTTAAGTTCATTACCACCTGTAACGGAT | Fragment B |
| C1-6448-F | GCGTTAATTAAAATGAACTTAAGCACCTATGCC | Fragment C  (SOE PCR) |
| C1-8979-R | TTGACACAGAGGTAATCGGGTCGCCAGAC |
| C2-8951-F | GTCTGGCGACCCGATTACCTCTGTGTCAA |
| C2-11945-R | CGGGGGAAAATGAAACCTCATGCTGGT |
| D-11865-F | TCGTTAATTAAGTTTCGGGCGCGCCAGAAAGGG | Fragment D |
| D-15425(SwaI)-R | **TTCGGCTTGGGATTT**AAATATGCATTTTTTTTTTTTTTTTTTTTT | Insert poly A tial |
| D-15464(SpeI)-R | CTCACTAGTAACGGCCGCCAGTGTGCTGGAA**TTCGGCTTGGGATTT** | Insert SpeI |
| Primers for the construction of site-directed mutations in plasmid pCMV-BB0907 | |  |
| BB0907-N(N15D)-F | GAAAAAGAAGGGGGATGGCCAGCCAGTC | Site-  directed mutagenesis |
| BB0907-N(N15D)-R | GACTGGCTGGCCATCCCCCTTCTTTTTC |
| BB0907-N(R46K)-F | GGGAAGAAAAATAAGAAGAAAAACCCGG |
| BB0907-N(R46K)-R | CCGGGTTTTTCTTCTTATTTTTCTTCCC |
